# Neighbourhood benthic configuration reveals hidden co-occurrence social diversity

**DOI:** 10.1098/rstb.2023.0174

**Published:** 2024-07-22

**Authors:** Stuart Kininmonth, Diana López Ferrando, Mikel Becerro

**Affiliations:** ^1^ Heron Island Research Station, University of Queensland, Gladstone, Queensland 4670, Australia; ^2^ Facultat de Biologia, Universitat de Barcelona, Avda. Diagonal 643, Barcelona 08028, Spain; ^3^ The BITES Laboratory, Center for Advanced Studies of Blanes (CEAB-CSIC), Access Cala S Francesc 14, Blanes, Girona 17300, Spain

**Keywords:** benthic, social diversity, Spanish coast, ERGM, graph theory

## Abstract

Ecological interactions among benthic communities are crucial for shaping marine ecosystems. Understanding these interactions is essential for predicting how ecosystems will respond to environmental changes, invasive species, and conservation management. However, determining the prevalence of species interactions at the community scale is challenging. To overcome this challenge, we employ tools from social network analysis, specifically exponential random graph modelling (ERGM). Our approach explores the relationships among animal and plant organisms within their neighbourhoods. Inspired by companion planting in agriculture, we use spatiotemporal co-occurrence as a measure of mixed species interaction. In other words, the variety of community interactions based on co-occurrence defines what we call ‘co-occurrence social diversity’. Our objective is to use ERGM to quantify the proportion of interactions at both the simple paired level and the more complex triangle level, enabling us to measure and compare co-occurrence social diversity. Applying our approach to the Spanish coastal zone across eight sites, five depths, and sunlit/shaded aspects, we discover that 80% of sessile communities, consisting of over a hundred species, exhibit co-occurrence social diversity, with 5% of species consistently forming associations with other species. These organism-level interactions probably have a significant impact on the overall character of the site.

This article is part of the theme issue ‘Connected interactions: enriching food web research by spatial and social interactions’.

## 1. Introduction

Ecological interactions among benthic communities play a vital role in shaping the structure and dynamics of marine ecosystems. Understanding these interactions is essential for predicting the responses of ecosystems to environmental changes, invasive species, and conservation management [1–3]. If an ecological partnership exists and one of the partners is suppressed, this can affect the broader ecological community [4,5]. Benthic organisms include many species, such as invertebrates, algae, corals, and microbes, which interact with each other and their surrounding habitats in complex ways [6].

Unfortunately, these interactions have been overlooked since traditional methods of estimating benthic biodiversity [7,8] focus on counting the number of species and individuals within a transect (alpha diversity) and then analysing the differences and trends across sites and regions [6,9]. Studies with a specific focus on interactions have concentrated on isolated pairwise relationships, such as mutualism [10] and competition [11–13], and between specific species [14,15]. Note that, more complex community network approaches have recently been conducted for Antarctica [4] and the Baltic Sea [5]. At a different scale, research has focused on the interaction between metapopulations [16,17], focusing on species persistence across the region [18–20]. The assumption when statistically analysing the presence/absence data is that the individual organisms captured in the survey are independent at a small spatiotemporal scale [21]. Blanchet *et al*. [22] demonstrate that inferring the co-occurrence of species from aggregated presence-absence data is problematic and that direct evidence of interaction is required.

Observing the interactions amongst the benthic neighbourhood, defined as the spatial arrangement and connectivity of benthic sessile and mobile organisms, is complicated by the propensity of the sessile organisms to colonize substrate and then grow vertically and horizontally within a closely spaced community and at different rates and times. Hence, the inference of interaction based on solely on spatiotemporal co-occurrences can result in the ‘gambit of the group’ assumption [23] for mobile animals and is difficult to disentangle from deliberate engagement. However, the science of companion planting in agriculture has matured with significant support for cross-species interactions, providing advantages to the mobile and sessile organisms involved [24,25]. Within this research domain, the co-occurring interaction of species, both trophic and non-trophic in origin [26,27], is firmly established [15,28–30] and, recent advances in network analysis have provided a new perspective to explore complex interactions within entire communities [5,27,31,32].

In this study, we propose a new perspective called ‘co-occurrence social diversity’ that measures the proportion of structural groups, such as dyads and triads, between organisms within an ecological setting [5]. These structural groups arise from network theory and are defined by their topology [32]. A dyad is a connection between two network entities (i.e. organisms) while a triad is defined as three entities connected in a closed triangle [33]. This diversity measure is based on ecological interactions directly observed in the neighbourhood, specifically those involving a shared common boundary. Co-occurrence social diversity, similar to functional diversity and species richness measures, is partially encapsulated in alpha diversity. However, co-occurrence social diversity focuses on preferential interactions to assess the structural variability across and within communities.

For mobile species, individuals can take advantage of the structural and functional characteristics of neighbouring species [34,35], with crabs, for example, using coral colonies [36] as shelter from other mobile predators during the day. Sessile species, on the other hand, rely on successful settlement followed by growth within an environmental and biotic niche [37]. The neighbourhood assemblage can involve negative, neutral, and positive interactions [6,38] between both mobile and sessile resident organisms [21,39]. Disturbance regimes can impact the community state, resulting in observed assemblages with an underlying degree of chaos [40].

The analytical framework to model community interactions is commonly based on graph theory [11,41] (also widely called network theory), and recent advances in statistical modelling for social networks [42,43] have advanced the application across disciplines [44]. Researchers have typically examined biological interactions in isolation, such as the number of connections or triangles observed within a network [45,46] and then inferred the process that might have resulted in the creation of that network. However, this approach ignores the dependency of the tie creation, which can lead to endogeneity [47]. In other words, the features observed in a network may be the result of edges that are created independent of an interaction process. What is required to disentangle the network of interactions is a model that can estimate the probability of edges and more complicated structures forming in a way that captures the dependencies across the network.

Constructing such a model to explain the formation of natural networks is not an easy task. The statistical analysis of a network is challenging since each linkage possibly creates a set of dependent subgraphs or motifs [48]. Adding a single edge to the network can create more complex structures, such as a triad in the immediate vicinity. However, this new triad may not have a biological explanation. In the field of social science, if participants identify the members of their friendship group they will often form triangles within the human friendship network and this indicates the likelihood of friends forming between mutual acquaintances [42,49]. Establishing the interactions in the biological systems requires close scrutiny in space and time. If we can construct a network of interactions across a community then we may struggle to describe the topology of the interactions using standard statistical methods [50]. Fortunately, the Hammersley-Clifford theorem has provided the theoretical foundations for constructing networks based on the probability of sub-structure occurrences, such as triads [51,52]. This theorem states that the probability of a particular network can be defined solely by the counts of subgraphs (triads etc. [53]). In other words, a network can be built from smaller micro-scale network structures with a certain probability of occurrence. Therefore, the construction of a network model depends on whether dyads and triads are formed for a specific reason (factor) or simply because of the likelihood of three vertices being connected. Hence, we are interested in understanding to what extent a secondary factor (possibly an ecological benefit) influences the probability of an edge being created.

One promising technique for this is the use of exponential random graph models (ERGMs or P*), which form a class of statistical models specifically designed for network analysis [50,53,54]. The goal of an ERGM is to identify the processes that influence the probability of edge creation. Logistic regression models are commonly used to describe these probability distributions and can be applied to networks [47]. Conceptually, ERGMs are similar to multivariate regression models, where the explanatory variables are represented by a set of network configurations, and the dependent variable is the network itself [5]. However, unlike multiple regression models that assume data independencey, ERGMs assume the network is formed through processes of interactions across the network [5,43,55].

While the number of applications of ERGM in ecology is limited, there is a growing acceptance of animal behaviour research using this social network analysis tool [35,44,56]. Insights into the disease prevalence in animals’ [57,58], parasite interactions with primates [59], socioecological systems in agricultural systems in Madagascar [60], insect population stability [61], dolphin social interactions [62], marine food web stability [5], primate partner selection [63], and crab social groupings [64] are some examples of the application of ERGM to ecologically related research. Interactions focused on plants or animals/plants that are analysed with ERGM have not yet been published, primarily owing to the restrictive conceptual framework of social engagement [56] for plants.

Investigating the configuration of the neighbourhood provides a valuable approach to uncovering hidden ecological interactions within benthic communities. While more investigations are required to understand the character and processes of the neighbourhood interactions, this approach can assist with the recognition of these interacting organisms. This research has implications for marine conservation and management, as it can inform strategies for preserving the integrity and biodiversity of benthic communities in the face of environmental change [5].

Our goal is to highlight how a social network analysis method can be used to describe the benthic character in terms of co-occurrence social diversity. This relies on directly observing neighbour-to-neighbour interactions at the organism scale and then translating this data to graph theory. The use of ERGM permits the estimation of the probabilities for the creation of key sub-structures such as dyads and triangles. This metric of the proportion of species associated with a network sub-structure can be used to compare sites despite similar counts of biodiversity. Hence community composition and key species interactions can be identified for further investigation.

## 2. Methods

### (a) Study sites

**Figure 1.**
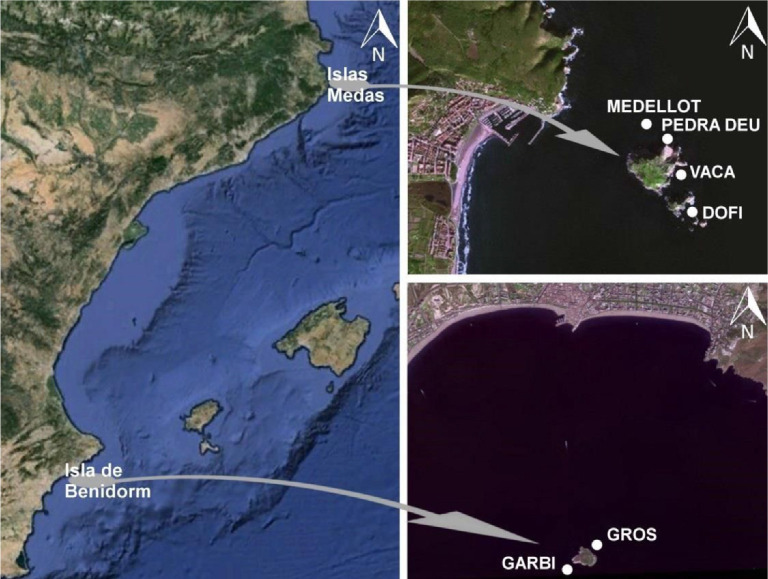
The sampling locations (also see table 1) off the coast of Spain.

The field data selection coincided with a programme to quantify changes in the sessile community along a depth gradient in multiple locations of two distant geographical areas [14,65]. The Spanish islands of Illes Medes and L'illa de Benidorm are located in the Mediterranean Sea and are nature reserves (figure 1). L'illa de Benidorm is located about 3.5 km from the coast, near Benidorm, and has been part of the Sierra Helada Natural Park since 2005. Illes Medes is an archipelago of seven islands about 0.85 km from L'Estartit. It has been protected since 1983 and became a National Protected Natural Park in 2010. These two locations were selected owing to their proximity to the coast, ease of sampling, rocky bottoms, and because they are island enclaves close to the coast.

### (b) Sampling design

During May and June 2010, six dive sites in Illes Medes and two dive sites in L'illa de Benidorm (table 1; figure 1) were selected. For each site, except Garbí and Gros owing to a lack of suitable substrate, a photophilous aspect was paired with a sciaphilous aspect. To capture the impact of depth on the community assemblages, each site was surveyed at different depths along the rocky reef wall at 2, 4, 7, 14, and 23 m deep. The sciaphilous aspects could not be located for the two sites in L'illa de Benidorm and only one set of transects at various depth profiles were photographed. Based on the continuous line transect method [14], photo quadrats (electronic supplementary material, figure S1) using a camera (Sony Cyber-shot Vario-Tessar 10.1 Mpx with a constant 45 cm distance measurement frame) covered a 5 m long transect for each depth profile. Approximately 25 overlapping images were captured per transect. Later, back in the laboratory, we identified every species (where possible) directly next to the photographed transect tape for each centimetre division so that 500 data points were recorded per transect. The final dataset contains 17 sites (table 1) with five depth transects, resulting in 85 sets of benthic data.

**Table 1.** Photo transects captured across eight sites with 17 surveys. (Each survey was done at the five depths of 2, 4, 7, 14 and 23 m. This resulted in 85 datasets.)

location	site	light exposure	label	surveys	latitude	longitude
Illes Medes	Medellot	sciaphilous and photophilous	ME.ES, ME.FO	2	42°3’7.41’’ N	3°13’18.43’’ E
Illes Medes	Pedra de Déu	sciaphilous and photophilous	PE.ES, PE.FO	2	42°3’0.94’’ N	3°13’28.52’’ E
Illes Medes	Vaca1	sciaphilous and photophilous	VA.ES, VA.FO	2	42°2’50.89’’ N	3°13’31.04’’ E
Illes Medes	Vaca2	sciaphilous and photophilous	VA2.E, VA2.F	2	42°2’50.89’’ N	3°13’31.04’’ E
Illes Medes	Dofi1	sciaphilous and photophilous	DO.ES, DO.FO	2	42°2’35.00’’ N	3°13’33.63’’ E
Illes Medes	Dofi2	sciaphilous and photophilous	DO2.E, DO2.F	2	42°2’35.00’’ N	3°13’33.63’’ E
L'illa de Benidorm	Garbí	photophilous	IB03., IB06.	2	38°30’5.49’’ N	0°7’57.10’’ W
L'illa de Benidorm	Gros	photophilous	IB02., IB05, IB07	3	38°30’13.46’’ N	0°7’49.32’’ W

### (c) Network creation

We translated the observational data from the field surveys into a series of networks based on noting the change in organism species type along the transect tape. Often, the single species is comprised of multiple colonies, but determining the internal boundaries is difficult in the field. For example, a transect with notation for every observation unit might look like AAAAABBBBCCAA… where A, B, and C are identifiable species for every centimetre and would translate to A(5)-–B(4), B(4)-–C(2), and C(2)-–A(2) edge list with size attributes (electronic supplementary material, figure S1).

To recognize the network theoretical model, we adopt the following notation and terminology [42,45]. A network *G*(*V,E*) is a finite set of vertices *V*, connected by edges *E*. Within *G*, *V_i_* and *V_j_* can represent two vertices which if connected can be described as *E*_*ij*_ = 1. If they are not connected then *E*_*ij*_ = 0. The probability of this edge *E*_*ij*_ forming is dependent on the structure of the network before a link between *i* and *j* is created. We assume that the edge formation is not a random process but instead is influenced by some set of variables [66]. The probabilities of adding each edge to create a network is now conditional on the pre-existing topology. The variables that influence this conditional probability can be described by the count of smaller sub-structures such as triads and other configurations. That is to say, the formation of the edges is conditionally linked to the vector of influencing variables ***Y***.

### (d) Exponential random graph model

ERGMs are established with foundations in spatial statistics and graph theory analysis [54,55,58] but have had limited applications in ecology [59]. In broad terms, the ERGM searches the observed network for explicitly stated sub-structures (dyads, triads, vertex attributes, etc.) and uses several stochastic techniques to estimate the probability of that structure (i.e., a triad) occurring given the presence of other structures (i.e., dyads). Hence sub-structures of a network can (and are predominantly) used as explanatory variables ***Y***. Once this has been completed, the ERGM can estimate the stated structures' significance in forming the observed network. Translating the logarithmic form of the marginal probability of an edge *E*_*ij*_ to the general form of the ERGM results in the following [67]:


(2.1) $$Pr\left(Y=y\right)=\frac{\exp\left[\sum_{A}\beta_{A}\;g_{A}\;\left(y\right)\right]}{k\left(\theta \right)},$$

where:

- *Y* is the matrix of all variables (often referred to as an adjacency matrix) such that the observed *y* is a particular realization of *Y* over all configuration types *A*;
-
$\beta_{A}$ is the parameter corresponding to the ***A*** configurations possible;
- *g*_*A*_(*y*) is the network statistic (i.e. probability of the number of edges, triangles, etc.) corresponding to type ***A***; and
- *k*(*θ*) is the normalizing quantity that represents the set of all possible networks ***Y*** [66]. Note that, this number is very large and a network with *n* vertices has a number of possible networks approximating as 2^*n*(*n* − 1)^. Estimating this normalizing constant requires estimation techniques such as maximum likelihood [66].

### (e) Network analysis

For this benthic survey set, we used the primary statistic to be the number of ‘edges’ for each vertex, which then translates to the likelihood of a vertex having a connection to another vertex. In ecological terms, this describes the role each species has within the community. A species with a high number of edges is highly interactive and central to the community functions. A species with a low edge count is on the peripheraly of the community processes. This statistic also captures the likelihood that two species will be interacting strongly in a dyad configuration.

**Figure 2.**
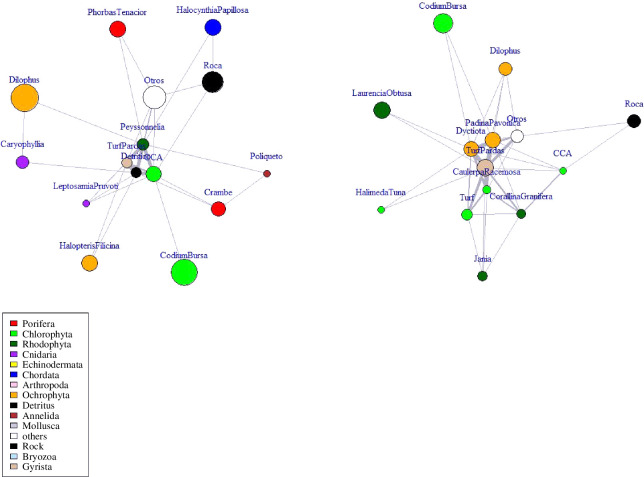
Network plots from the Illes Medes, Dofi2 site at 23 m in shaded aspect (left) and the L'illa de Benidorm, Garbi site at 7 m full sunlight. The size of the vertex represents the mean size of the organism while the line width represents the number of observations of an organism-to-organism adjacency. The vertex labels are the species shorthand and fully described in the electronic supplementary material, table S1. The phylum associated with the species is shown in different vertex colours and displayed in the legend.

The next statistic of interest for this model is the likelihood of being in a triad. Currently, very few studies have focused on the field observation of triads, primarily owing to the difficulty of directly observing the series of interactions. Yletyinen *et al*. [5] used trophic interactions to describe triads and more complex structures in the marine environment but this was based on connecting individual interactions to create sub-structures of varying configurations rather than observing the entire set of interactions. More complicated sub-structures (see fig. 1 in [5]) were not used in our research simply owing to the difficulty in translating to ecological processes at this stage. Since the estimation of the probability for triangle creation across the entire network can lead to model degeneration [47], we use the geometrically-weighted edgewise shared partnerships (GWESP) statistic [50]. This algorithm has the option of a fixed decay parameter (to limit the size of the network being analysed) or is alternatively fitted as a curved exponential-family model [55]. Important to note is that the multiple edges present in the networks (see figure 2) are essentially ignored by ERGM but are captured in the vertices covariates. The next statistics used in this model were based on the quantitative attribute of organism size total (cm) and count for each transect. Additionally, the species names were used as a factor and designed to seek the preference of particular species to influence the mean degree distribution. Owing to the compounding dependent terms in the model, the ERGM uses Markov chain Monte Carlo (MCMC) algorithms with a Metropolis-Hastings sampler to estimate the parameters [55]. We used the R packages *network* [68], *ergm* [55,66], *Intergraph* [69], and *igraph* [70].

Two ERGMs were computed for each network that focused on different aspects of the networks. Model 1 used the formulae of edges, GWESP with covariates *size* and *count* (in R: G~edges +gwesp(0.25, fixed = TRUE)+nodecov(‘size’)+nodecov(‘count’)). The second model was based on edges and species names (in R: G~edges +nodefactor(’vertex.names’)). Given the many species names, this model split was required owing to model degeneracy issues. Model degeneration can be problematic when too many parameters are combined and the generated networks can be very dense or sparse. Even with the model split, 17 networks did not converge on model 1, and a restricted model 1A was applied without the GWESP (in R: G~edges + nodecov(‘size’)+nodecov(‘count’)). Critically the ERGM generates model coefficients and a *z* **-**score that highlights how various components such as edge preferences influence the creation of simulated networks. Positive coefficients show how a new edge is best placed in the network to support the associated sub-structure (dyad or triad) or even covariate (species names, size). Negative coefficients indicate a preference to place the new edge away from the creation of sub-structures and co-variates [66].

The success of the estimates to capture the formation of the observed network is measured via goodness-of-fit (GoF) simulations with standard statistical tools such as Akaike and Bayesian information criterion (AIC and BIC) [43]. The ERGM can generate multiple networks which when they are aligned optimally with the observed network, the parameters (probability of edges, triads, etc.) can be considered statistically valid influences on the observed network creation [71]. Hence, the primary advantages of the ERGM are the capacity to generate a novel network of the same statistical properties as the observed network with a clear link to the influential processes.

The primary disadvantages are the capacity for model degeneration, especially in complex large networks [72] where the algorithm struggles to converge [73]. The stochastic variability of the network creation makes comparisons between systems difficult. Another ecological limitation is the data requirements combined with modelling expertise in this field. Random forest analysis [74] determined the influence of the site, depth, aspect, and network size on the probability of triangles forming (GWESP) in the networks based on the GoF *p*-values.

## 3. Results

We generated 85 networks (using *igraph* [70,75] in R [76]) while observing 92 species in Illes Medes and 39 species in L'illa de Benidorm for a total species count of 101 (electronic supplementary material, table S1). Each network was undirected, contained multiple edges, and had no isolated vertices (figure 2). The networks had a mean of 16.64 vertices (minimum: 14, maximum: 27) linked by a mean of 190.1 edges (minimum: 148, maximum: 395).

### (a) Statistical evaluation of the exponential random graph model

**Figure 3.**
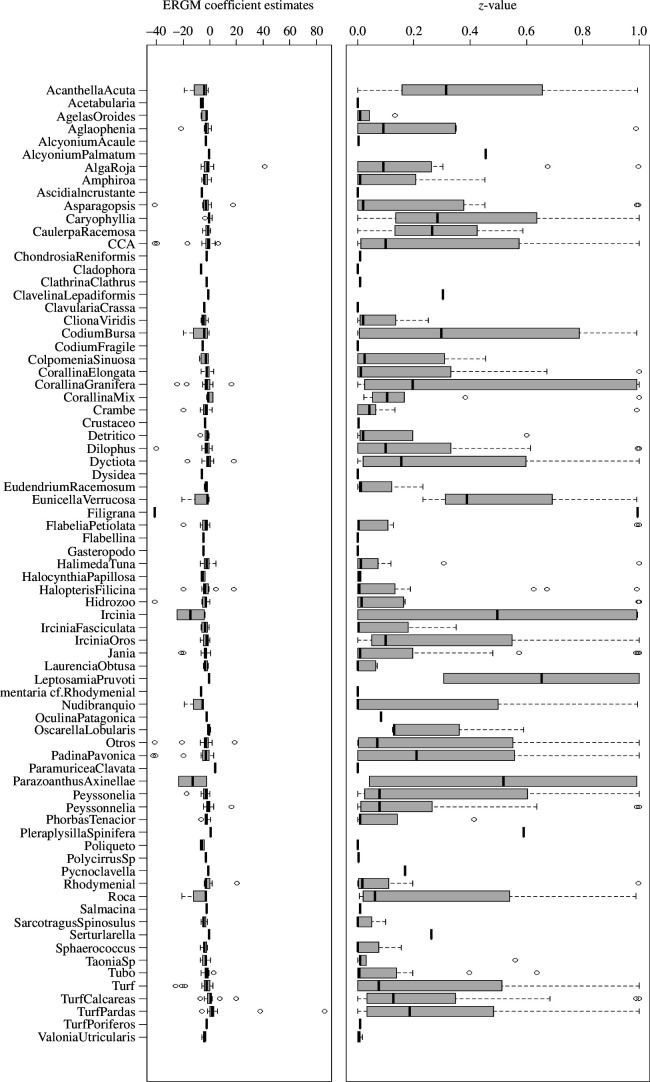
The ERGM model 2 coefficient estimates (left) and the associated *z*-values (right) for the species where organisms occur more than five times across the 85 transects. The coefficient estimates oscillate around 0 where the positive numbers indicate the likelihood for preferential attachment in the ERGM model. Negative coefficient estimates indicate that the species did not form dyads commonly and that the ERGM model performs best when placing new vertices elsewhere in the network. The smaller *z*-value indicates a more significant contribution to the formation of the ERGM estimations. The *y*-axis labels are the species shorthand and fully described in the electronic supplementary material, table S1.

**Figure 4.**
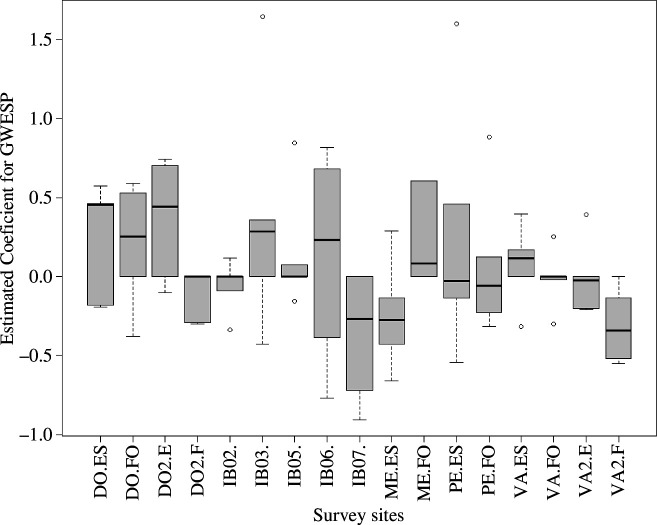
Estimated coefficients for GWESP from the ERGM across all sites (table 1). Coefficients greater than zero indicate a preference for increased number of triads while values less than zero indicate an aversion for triads. In other words, sites like IB07 have networks that are modelled with less influence of triads than DO2.E where the inclusion of triads is essential for model development. Sites like VA.FO are essentially random in composition in that no influences are used to construct the simulated models.

Our primary question to evaluate here was the presence of statistically significant sub-structures across the survey sites, light exposure and depths. Hence the ERGM algorithms (models 1, 2, 1A) were conducted separately on the 85 networks. The MCMC diagnostics (electronic supplementary material, figure S2) for the converged ERGM models were examined and showed consistent support for the model construction. The models 2 and 1A that did not have triad dependency included did not run MCMC. The Monte Carlo maximum likelihood estimations follow a logistic regression format where the ERGM provides an estimate and standard error, and a *z*-value is calculated. The estimated parameters were used in a GoF algorithm, which created 100 simulated networks for each ERGM model, and then compared with the observed network (for the 85 models: edge *p*-value: mean = 0.99, s.d. = 0.03, GWESP *p*-value: mean = 0.91, s.d. = 0.07, size *p*-value: mean = 0.9, s.d. = 0.06, count *p*-value: mean = 0.91, s.d. = 0.06; electronic supplementary material, figure S3). The interpretation of these values highlights that the edge distribution and triad formation influence the formation of the majority (68 out of 85) of networks. Model 2 includes the individual species as factors and is evaluated by the coefficient estimation and the *z*-value (figure 3; est:. mean: −3.39, s.d.: 8.36, *z*-value: mean = 0.21, s.d. = 0.33). Figure 3 highlights that there is a high variation in species for preferential connections. Site identity contributed significantly to the network formation (figure 4), which may relate to disturbance history.

### (b) Ecological interpretation of the exponential random graph model

While most of the species observed showed no significant edge preferences (figure 3), five species demonstrated significant homophily (positive ERGM coefficients and low *z*-score) towards other species; *Aglaophenia* sp. (*n* = 21, median *z*-value = 0), *Colpomenia sinuosa* (*n* = 46, median *z*-value = 0), *Ellisolandia elongata* (*n* = 46, median *z*-value = 0.01), *Flabellina affinis* (*n* = 43, median *z*-value = 0.01), and *Jania rubens* (*n* = 44, median *z*-value = 0.01). Several category groups (electronic supplementary material, table S1) such as AlgaRoja (category 1, *n* = 24, median *z*-value = 0.02), CCA (category 2, *n* = 74, median *z*-value = 0.04) and Dyctiota (category 5, *n* = 70, median *z*-value = 0.09) were also significant in their edge preferences (figure 3). The propensity of some species to be found in triangular structures highlighted the potential interactions present (for example see the electronic supplementary material, figure S5, for *Aglaophenia* sp.).

While individual species and their observed role in the community network is instructive towards the co-occurrence social diversity, the emergence of the character of the phylum groupings (electronic supplementary material, table S1) is instructive. Based on the aggregation of the ERGM coefficients and the *z* scores, each phylum shows distinct roles in the community assemblage (figure 2; electronic supplementary material, S6). Not surprisingly, the Porifera species (sponges) tended to generate bioactive agents that inhibit neighbour growth and this is shown by the negative values of the coefficients (mean = −4.1) and low z-score (mean = 0.2). Similarly the species belonging to Chlorophyta phylum (green algae) showed an aversion to neighbour groups (coefficient mean = −4.04 and *z*-score mean = 0.21) possibly as a response to an aversion for shading by neighbours. By contrast, the Gyrista phylum that is composed of three diverse groups of protists showed a preference for neighbour structures (coefficient mean =+4.09 and *z*-score mean = 0.31) whilst noting that for the Spanish sites, this was limited to the observation of brown algae. This phylum appeared to be commonly linked to dyads and triad development. The Rhodophyta phylum is currently in a state of flux taxonomically but the species contained within this grouping showed a diversity of neighbourhood configurations (coefficient mean = −3.86 and *z*-score mean = 0.22) with no obvious trends in site or depth. The Ochrophyta grouping which contains the photosynthetic stramenopiles also showed a high diversity of structural configurations (coefficient mean = −3.81 and *z*-score mean = 0.23). Overall when examined across the survey sites only a small number of phylum groups had a preference for being in dyads or triads (coefficient mean = −3.06 and s.d. = 10.3).

Location-based factors such as site identity, depth, and aspect combined with network vertex and edge number were evaluated as an influence of the GoF *p*‐value. This included the GWESP statistic (within the model 1 algorithm) using a relative influence measure within a random forest framework. This technique showed that site identity (60.2%) was an essential determinant in forming triangles for the networks (figure 4; electronic supplementary material, figure S7). The subsequent relative influences were the number of edges at 20.9%, the depth of the transect at 9.7%, and the number of vertices at 8.7%. The univariate partial dependency plots in the electronic supplementary material, figure S7, highlight that as the number of edges or the depth increases then the ERGM improved in replicating the observed network. This implies that vertices that are well connected or are located at greater depth are able to be modelled more accurately with ERGM.

## 4. Discussion

The examination of 85 communities from a Spanish benthic environment shows both selective mixing and triad formation operate across a wide range of depth, site, and light intensity settings. Using a social network analysis framework, in particular ERGM, we showed that 30 species (electronic supplementary material, table S1) out of 101 (including two benthic general categories) were consistently found (*z*-value < 0.05) to have preferential attachment (figure 3). By contrast, many others (*n* = 71) did not show neighbour preferences. The biological reasons behind the positive and negative preferences are unknown but could relate to chemical, biological or structural interactions [14,77]. Potentially, trait-based influences could determine the social interactions [78]. The presence of a benthic organism along the survey transect is assumed to be an indicator of settlement and growth success, influenced by social interactions. Importantly, the comparison of the different survey sites can be enhanced through the measurement of the co-occurrence social diversity as shown in figure 4. Sites that have similar biodiversity measures can demonstrate different community structure and this may impact on the resilience of the site. However, without knowing the disturbance history or the capacity of the benthic community to reach a climatic state [5], the observation of co-occurrence social diversity only indicates potential interactions and additional *in situ* observations are needed to determine the processes involved [14].

A closer examination of the species involved in the formation of triangular motifs highlights that sub-structures were common. For example, the *Aglaophenia* sp. (Linnaeus, 1758) genus of hydrozoans in the family Aglaopheniidae is one group that showed preferential attachment (electronic supplementary material, table S1). This group have a feather -like morphology when sessile. We identified common neighbours for this group and this includes *Corallina elongata* (J. Ellis & Solander 1786), *Jania rubens* (J. V. Lamouroux 1816), *Corallina granifera* (J. Ellis & Solander 1786) and *Dictyota* sp. ( J. V. Lamouroux 1809). All of these organisms have short fronds commonly less than 5 cm in length which might provide habitat for small invertebrates and molluscs as well as create microclimates for water flow. Another possibility is the shared allelochemical transmissions, especially for sponges [77,79]. For some species like *Crambe crambe*, a Poecilosclerid sponge, the production of bioactivity agents can inhibit the growth neighbouring species [14]. These bioactive metabolites will act in a very limited spatial range and so the neighbour boundary is a key interaction measure [79,80]. Hence the ERGM will model the network including this species with a reduced probability of attachment preference (shown by a negative ERGM coefficient and a low *z*-score; figure 3). However, the benefits of the associations for many other species remain unknown at this stage.

The previous biodiversity measures that aggregate the observations along a transect [81] may be ignoring the organism-to-organism dependencies. These interactions for sessile organisms are cryptic but may also involve mobile species, such as invertebrates, that use the structural shelter of the various benthic organisms. Previous research has identified interacting pairs of organisms such as crab/coral [36] and clownfish/anemone [82] but commonly assume that competition for benthic substrate and light dominate the community assemblage. This benthic research highlights that local scale interactions may be shaping community diversity more than was previously thought. Previous approaches to analysing interaction outcomes in epibenthic communities [77] focused on the overgrowth and growth inhibition at contact edges. Competing individuals or colonies were examined for fine scale interactions (e.g. [83,84]). These studies however had a limited scale of interaction and were not able to address mechanisms acting at a larger scale [14].

The ERGM method is able to disentangle the complex networks of community relationships into a series of local scale interactions. However, the application of social network analysis, and in particular, the use of ERGMs, is diminished in the field of ecology. This is most likely owing to the availability of suitable data but could also relate to the lack of expertise in this area. The statistical power of ERGM makes this approach attractive [85], especially in the awkward methodological process of generating networks for comparison. With the overt inclusion of dependencies in the network modelling frameworks, the ability of ERGM to generate significance values in the form of *z*-values and AIC/BIC is important. By contrast, using standard statistical methods, with their associated assumptions of independence, is problematic and potentially misleading. In particular, the GoF approach (electronic supplementary material, figure S3), where the ERGM can simulate a set of comparable networks as a measure of the structural foundation of the observed network, is powerful indeed. This is notwithstanding the model degeneracy issue [86] where the parameter space estimation fails. Seventeen of the observed networks (electronic supplementary material, figure S4) could only be estimated with edge distribution (essentially degree density) combined with two vertice covariates, while the rest of the networks used the dyad dependencies with triad measurement along with vertice covariates. Continued algorithmic development in this field may help resolve this conundrum [73,87]. Despite this, the powerful ERGM technique to unravel a highly interdependent mathematical structure like a network and then confidently rebuild a novel one based solely on parameter estimates will ensure the continued development of ERGM in this field [88].

Obtaining data that highlights interactions rather than co-occurrence presence-absence data is essential for ecological studies [22]. Yletyinen *et al*. [5] highlighted the use of ERGM within a trophic network setting and with established interactions. However, many ecological systems at a variety of organismal scales remain poorly studied. Here, we offer a statistical mechanism to explore the hidden interactions, especially from a non-trophic context. Focusing on every potential interaction combination between species, especially in areas of high biodiversity, would be prohibitive and hence this tool is able to constrict the research focus to a small number of interacting species.

This study is based on linear transects, and there is a capacity to increase the dimensions of the surveys to both two and three dimensions. This type of data is becoming available with automatic image compilation, especially in the reconstruction of the benthic relief [37]. Possibly, four-dimensional benthic data can be obtained to solve the issues of community interactions through time. The other limitation is the scale of the surveys which, in this example, were limited to organisms close to 1 cm width or larger. Owing to the time limitations of underwater surveys, especially at depth, the data must be collected rapidly and with instrument limitations. The same scale issue applies to mobile organisms, but developments in the field of underwater robotics may overcome these challenges.

The arrangement of the neighbourhood can have significant implications for ecological interactions and community dynamics [89,90]. For instance, the spatial arrangement of different habitat types, such as rocky reefs, seagrass beds or sandy bottoms, can influence the distribution and abundance of interacting species [15,27,91–93]. These habitat types may act as ecological filters, promoting or hindering the establishment and survival of certain species. Early research focused on the co-occurrence of species across a gradient as a model for interaction [92] or as the probabilistic association [28,94]. The processes of colonization and survival for each species operate within a co-dependent community environment that can radically shift from one regime to another when sufficiently disturbed [5].

These cryptic interactions leading to a high social diversity may be critical to the health and function of a community. Critical issues such as species invasions and push/press disturbances [95] can reshape the community neighbourhood and possibly diminish the resilience of the benthic habitat. Understanding that the role of each species is more than a percentage along a transect may be vital to understanding the ecological function. Restoration ecology must be aligned to the combination of alpha and social diversity to be effective; otherwise, it may introduce another form of disturbance.

## Data Availability

Data are available on Dryad [96]. Data is also available in the electronic supplementary material [97].
